# Hypothalamic-Ovarian axis and Adiposity Relationship in Polycystic Ovary Syndrome: Physiopathology and Therapeutic Options for the Management of Metabolic and Inflammatory Aspects

**DOI:** 10.1007/s13679-023-00531-2

**Published:** 2024-01-03

**Authors:** Maria Serena Lonardo, Nunzia Cacciapuoti, Bruna Guida, Mariana Di Lorenzo, Martina Chiurazzi, Simona Damiano, Ciro Menale

**Affiliations:** Department of Clinical Medicine and Surgery, Physiology Nutrition Unit, Federico II University of Naples, Via Sergio Pansini 5, 80131 Napoli, Italy

**Keywords:** Adiposity, H-P-O axis, PCOS, Low-grade chronic inflammation, Hyperleptinemia, Tailored therapy

## Abstract

**Purpose of Review:**

The goal of the present review is to address the main adiposity-related alterations in Polycystic Ovary Syndrome (PCOS) focusing on hypothalamic-pituitary-ovarian (H-P-O) axis and to provide an overview of nutraceutical and pharmacological therapeutic strategies.

**Recent Findings:**

Female reproduction is a complex and delicate interplay between neuroendocrine signals involving the H-P-O axis. Elements that disrupt the balance of these interactions can lead to metabolic and reproductive disorders, such as PCOS. This disorder includes menstrual, metabolic, and biochemical abnormalities as well as hyperandrogenism, oligo-anovulatory menstrual cycles, insulin resistance, and hyperleptinemia which share an inflammatory state with other chronic diseases. Moreover, as in a self-feeding cycle, high androgen levels in PCOS lead to visceral fat deposition, resulting in insulin resistance and hyperinsulinemia, further stimulating ovarian and adrenal androgen production.

In fact, regardless of age and BMI, women with PCOS have more adipose tissue and less lean mass than healthy women. Excessive adiposity, especially visceral adiposity, is capable of affecting female reproduction through direct mechanisms compromising the luteal phase, and indirect mechanisms as metabolic alterations able to affect the function of the H-P-O axis.

**Summary:**

The intricate crosstalk between adiposity, inflammatory status and H-P-O axis function contributes to the main adiposity-related alterations in PCOS, and alongside currently available hormonal treatments, nutraceutical and pharmacological therapeutic strategies can be exploited to treat these alterations, in order to enable a more comprehensive synergistic and tailored treatment.

## Introduction

The physiology of female reproduction is a complex and delicate interplay between neuroendocrine signals involving the hypothalamic-pituitary-ovarian (H-P-O) axis. Elements that disturb the balance of these interactions can cause metabolic and reproductive disorders.

Alterations in the proper functioning of the H-P-O axis signals are frequent in overweight or obesity [1].

Leaving aside the role that excessive adiposity can play in the delicate moment of the onset of puberty [2], further repercussions can be certainly seen in adulthood, with an impaired reproductive sphere [3]. The presence of adiposity can interfere with the physiological functioning of the H-P-O axis both directly, by disrupting the pituitary luteinizing hormone (LH) pulse amplitude and its average release without changing its frequency and determining an impaired luteal phase [4], either indirectly, through major adiposity-related alterations, such as insulin and leptin resistance and Low-Grade Chronic Inflammation (LGCI) [4].

One of the most prevalent disorders of the reproductive age encompassing all these alterations with a close link between H-P-O axis and metabolic perturbations is the Polycystic Ovary Syndrome (PCOS).

**Fig. 1:**
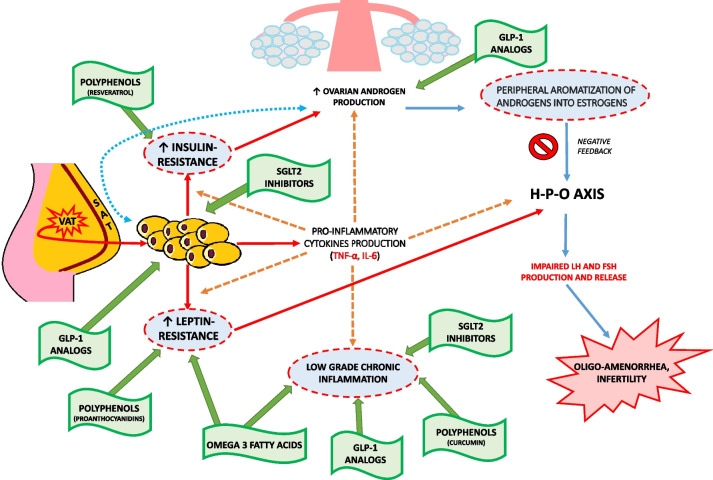
PCOS physiopathology and therapeutic options for the management of metabolic and inflammatory aspects. VAT: Visceral Adipose Tissue; SAT: Subcutaneous Adipose Tissue; Red arrows: Adiposity pattern; Orange dotted arrows: Low-grade chronic inflammation pattern; Blue arrows: Hyperandrogenism pattern; Green arrows: Therapeutic options

The purpose of this review is to address the major alterations related to adiposity and its interaction with the H-P-O axis in PCOS, focusing on insulin resistance (IR), leptin resistance, and LGCI. Furthermore, our aim goes beyond the consideration of currently available hormonal treatments, but aims to provide an overview of nutraceutical and/or pharmacological therapeutic strategies for the adjuvant treatment of inflammation and metabolic alterations typical of PCOS, in order to enable a more comprehensive and synergistic treatment. Substances such as Polyunsatured Fatty Acids (PUFAs) and Polyphenols will be reviewed on the one hand and anti-inflammatory molecules, Glucagon-like Peptide 1 (GLP-1) analogs, and Sodium-Glucose Transport Protein 2 (SGLT2) inhibitors on the other (Fig. 1).

### H-P-O axis and Polycystic Ovary Syndrome

The H-P-O axis is responsible for allowing procreation by cyclically producing gonadotropic and steroid hormones [5].

Starting from the hypothalamus, gonadotropin-releasing hormone (GnRH) triggers the release by the pituitary gland of the two gonadotropins, follicle-stimulating hormone (FSH) and luteinizing hormone (LH), which, in turn, exert their action peripherally on the ovaries to stimulate follicular growth and lead to the production of estradiol and, after ovulation, progesterone [1]. When estradiol reaches a constant threshold in terms of concentration (generally greater than 200 pg/mL) and duration, for at least 24–48 hours, it provides positive feedback that increases the frequency and decreases the amplitude of GnRH pulses, thus triggering the increased pituitary release of LH for ovulation [1]. Then, through a negative feedback mechanism, estradiol, together with inhibin B, acts on the hypothalamus suppressing FSH release [6]. One of the most common endocrine and metabolic disorders of the reproductive age in women is the PCOS which is considered part of ovulation disorders involving dysfunction of the H-P-O axis and is often accompanied by alterations in glucose metabolism, hyperinsulinaemia, hyperleptinemia, cardiovascular diseases, infertility and psychiatric disorders [5, 7, 8••].

The diagnosis of PCOS is made by the presence of at least 2 of the Rotterdam criteria [9] including oligo-ovulation or anovulation, clinical and/or biochemical signs of hyperandrogenism and polycystic appearance of the ovaries on ultrasound examination. The etiopathogenesis of PCOS is still not entirely clear, since there are numerous hypotheses but none of them are really established and indisputable. Genetic factors or multiple environmental drivers such as lifestyle, obesity, other endocrinological disorders such as dysthyroidism or impaired adrenal function, and endocrine disruptors are mentioned among the possible causes of PCOS [9].

In particular, as an hallmarks of the disease, in comparative studies between women with PCOS and healthy controls it has been described an impairment (much more often a decrease) in LH pulse amplitude not always accompanied by an increase in LH pulse frequency, with similar FSH levels between the two groups [4]. This dysbalancing in LH pulse amplitude and frequency would appear to be caused by an alteration in the pulsatile secretion of GnRH by the hypothalamus [5].

The primum movens of altered GnRH secretion is not yet entirely clear and the complexity of its regulation is directly proportional to the complexity of the mechanism. It has been observed that insulin is one of the elements that can disrupt the proper functioning of the H-P-O axis, being able to increase the frequency and amplitude of the secretion of GnRH and LH pulses through the upregulation of GnRH gene expression in hypothalamic GnRH neurons by activation of the MAPK pathway [10].

The persistently rapid pulsatility of GnRH, which promotes greater pituitary synthesis of LH than that of FSH resulting in increased LH concentrations and LH:FSH ratio (normal value <2) is typical of this disorder. This represents a physiological stimulus for androgen synthesis by theca cells and the cause of hyperandrogenism in PCOS [11] because in the ovaries, LH dose-dependently regulates the activity of cytochrome P450c17, the enzyme that limits the rate of sex steroid synthesis in theca cells. But, as in a vicious circle, androgens themselves have been shown to increase the frequency of GnRH pulses through inhibition of negative feedback from sex steroids on LH secretion, which eventually leads to increased LH and androgen levels [12].

**Fig. 2:**
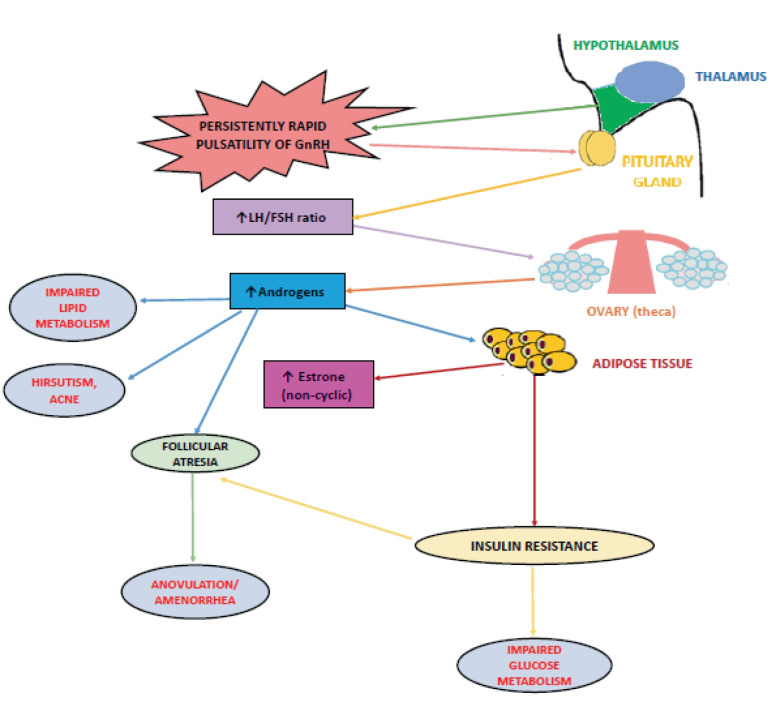
Impaired hypothalamic GnRH secretion and its main physiological and metabolic consequences in PCOS

Among the most important metabolic consequences of hyperandrogenism in PCOS is reported an altered body fat distribution, as excess androgen masculinizes the fat distribution pattern, promoting visceral fat accumulation and increasing its thickness [13] (Fig. 2).

All this leads to harmful metabolic consequences for PCOS patients, as increased visceral adiposity is a risk factor for the development of metabolic syndrome and cardiovascular diseases [14].

### Adiposity and Alterations of the H-P-O axis

Although the underlying pathological mechanisms are not exactly known, the association between obesity, particularly visceral obesity, and ovulatory disorders secondary to neuroendocrine alterations of the H-P-O axis is now well established [15].

Women with a surplus of visceral adipose tissue also have increased ovarian androgen production, likely caused by elevated insulin levels that often occur with obesity [16]. The exceeding overactive androgens are aromatized into estrogens at high rates in the periphery, causing negative feedback on the H-P-O axis and affecting gonadotropin production [17]. Moreover, as in a self-feeding cycle, high androgen levels in PCOS lead to visceral fat deposition, resulting in IR and hyperinsulinemia, further stimulating ovarian and adrenal androgen production. This turns into menstrual abnormalities and ovulatory dysfunction [18].

It has also been shown in several studies that women with PCOS have significantly higher amounts of body fat and less lean body mass than controls with the same age, weight and Body Mass Index (BMI). A similar situation is found in a murine model of PCOS, in which affected mice had a higher proportion of fat deposition from 6 months of age and a higher rate of metabolic and reproductive abnormalities than healthy controls [19].

The main negative effects of over-adiposity on the H-P-O axis are exerted directly, through a decreased pituitary LH pulse amplitude and mean LH release without changing its frequency, impairing luteal phase [4], or indirectly mainly through metabolic changes coexisting with obesity such as hyperinsulinemia and hyperleptinemia, that exerts pro-inflammatory activities, stimulating immune cells and also fueling insulin resistance [20] and LGCI. Indeed, increased body fat is often associated with visceral adiposity and IR, allowing adipose tissue to be identified as a source of cytokines [21] causing LGCI and dysovulatory infertility.

### Low-Grade Chronic Inflammation

As mentioned above, although PCOS recognizes a multifactorial etiology, one of the key players involved in its pathogenesis is certainly LGCI, which, in this case, acts at both the systemic and ovarian levels and would appear to play a cause-and-effect role in the disease, representing an important link to the metabolic perturbations that accompany PCOS [8••].

In the framework of systemic inflammation, previous studies have reported that significantly higher concentrations of inflammatory cells with increased whole leukocyte lineage and dysregulation between regulatory T cells and T cells have been detected in the peripheral blood of PCOS patients [22]. In several studies, in addition to alteration in leukocyte counts, high levels of pro-inflammatory molecules, such as Interleukin-6 (IL-6), Tumor Necrosis Factor-α (TNF-α), C-reactive protein (CRP), high-sensitivity CRP, and low levels of anti-inflammatory molecules namely Transforming Growth Factor-β (TGF-β) and Interleukin-10 (IL-10) have been found in both serum and follicular fluid of women with PCOS as compared with healthy controls [23, 24].

In particular, it has been described that IL-6, together with TNF-α, might be involved in this process, all the more so in the presence of PCOS-related metabolic perturbations, although the data in the literature in this regard are inconsistent [25]**.** Indeed, in some cases, in normal weight or slightly overweight women with PCOS, IL-6 and TNF- α levels were found to be elevated compared to both healthy and obese control women [26–29]**,** suggesting that increased circulating pro-inflammatory cytokines might be a feature of the PCOS pathology.

On the other hand, some other studies have associated the pro-inflammatory profile that occurs in PCOS [25, 30] with adiposity or obesity that could be indicated as the major determinant of serum inflammatory markers [31]. Furthermore, in another work, IL-6 and TNF-α circulating levels have been demonstrated to positively correlate with insulin resistance in obese women with PCOS [32].

Increased circulating levels of IL-6 lead to the activation of a subset of pro-inflammatory T-helper 17 (Th17) cells that, by increasing IL-21 expression, stimulate the release of the inflammatory cytokines IL-17A and IL-17F. TNF-α, at the same time, has the ability to directly stimulate the expression of IL-6 and cooperate with IL-23 to promote the expression of IL-17A [32]. IL-17A and IL-17F might be involved in the onset of the inflammatory profile in PCOS possibly by acting in synergy with TNF-α leading to the increase of IL6 production and contributing to an inflammation-related oxidative stress [32].

IL-6 also regulates CRP synthesis in the liver and is closely associated IR, obesity, cardiovascular disease [31] and increased androgen levels [33]. This increase in circulating pro-inflammatory cytokines well describe the systemic inflammation state driven by PCOS, and influenced by the associated metabolic dysfunction. At the local level, although mild ovarian inflammation is physiological in folliculogenesis during ovulation, when excessive, it can cause defects in oocyte quality, oligo-anovulation, and therefore cause infertility [34].

In a study by Xiong et. Al, by immunohistochemical staining of the ovaries of women with PCOS, an infiltration of large numbers of macrophages and lymphocytes was demonstrated, consistent with a condition of LGCI [35].

Ghowsi et al. demonstrates, in rats with PCOS, a higher representation of visceral fat and percentage of total fat compared with controls and higher mRNA expression of IL-6 and TNF-α within the visceral adipose tissue [36].

In fact, an important site of production of these inflammatory cytokines is precisely the adipose tissue [37] and in particular that with abdominal localization.

Furthermore, TNF-α plays a role in the pathogenesis of obesity-associated IR by blocking insulin receptor tyrosine kinase phosphorylation [38]. It also impairs glucose transport by reducing the activity of insulin-induced glucose transporter type 4 (GLUT-4) [39].

The above observations reinforce the idea that systemic and local inflammation links obesity, insulin resistance, and as a consequence, diabetes, which are very frequent metabolic alterations in PCOS [40], although further investigation is needed both at basic and clinical levels to dissect the cellular and molecular role of the inflammatory molecule in PCOS.

### Insulin-Resistance

Insulin is a 51-amino acid protein produced in pancreatic β-cells. It is the prototypical hypoglycemic molecule and promotes glucose uptake by insulin-sensitive organs and tissues. Given its hypoglycemic role, its secretion is stimulated by plasma levels of glucose produced following ingestion of food or, directly, by specific amino acid [41].

Conditions such as obesity or PCOS itself are associated with hyperinsulinemia and IR when, despite normal pancreatic function, the insulin produced fails to carry out its action and accumulates in the plasma [42].

IR has been described to be intrinsic to PCOS, independently of adiposity [43].

A possible mechanism for IR in women with PCOS appears to be related to excessive serine phosphorylation of the insulin receptor. Specifically, serine phosphorylation of the insulin receptor substrate-1 (IRS1) and insulin receptor substrate-2 (IRS2), leads to inhibition of insulin signaling. Furthermore, the auto-phosphorylation of insulin receptor induces a decreased expression of GLUT-4, which is the insulin-sensitive glucose transport protein. Hyperinsulinemia results in an increased risk for many diseases including, type 2 diabetes (T2D), hypertension, dyslipidemia, endothelial dysfunction (ED), atherosclerosis, and cardiovascular diseases [44]. Hyperinsulinemia also can cause reduced production of Sex Hormone Binding Globulin (SHBG) by the liver and, consequently, leads to increased levels of circulating steroid hormones, such as estrogen and androgens [45]. A possible mechanism leading to increased androgen levels comes through the binding of insulin to its receptors on theca cells, inducing an increase in LH activity at the ovarian level, which, in turn, stimulates androgen production and secretion [46]. So much so that in PCOS, hyperinsulinemia is often associated with high LH levels and biochemical hyperandrogenism, which, in turn, triggers adipose tissue dysfunction [47]. Although insulin resistance is associated with hyperandrogenism and anovulation, an excess of androgens is recognized among the possible causes of insulin resistance in PCOS [48].

Beside peripherally, insulin can also contribute to the development of PCOS through its actions at the central level. Indeed, insulin can influence the activity of hypothalamic Proopiomelanocortin (POMC) producing neurons in the arcuate nucleus that present insulin receptors, as well as leptin receptors [49]. In fact, transgenic mice lacking the insulin receptor and the leptin receptor in POMC neurons have been shown to induce the PCOS phenotype, indicating that insulin and leptin may be potent regulators of POMC neurons, further contributing to the development of PCOS [19].

Thus, IR, hyperandrogenism, hyperleptinemia, LGCI and adipose tissue hypertrophy and dysfunction may cooperate in the vicious cycle that contributes to the etiopathogenesis of PCOS [50].

In addition, we should taking into account that in PCOS, IR can induce an inflammatory response that increases nuclear factor-κB (NF-κB) activation, oxidative stress and altered release of TNF from circulating mononuclear cells (MNCs). It has been clarified that oxidative stress has an important role in LGCI and can be significantly augmented in PCOS by expression of pro-inflammatory cytokines [51]. Moreover, considering insulin also as an adipogenic factor, by increasing body fat mass stimulates leptin expression and secretion. In turn, leptin inhibits pancreatic β-cell functions through direct and indirect actions of central neuronal pathways [52].

### Leptin-Resistance

Leptin, first identified in 1994, belongs to the adipokine family and is a 16 kDa peptide hormone encoded by ‘*ob*’ gene [34]. This hormone plays a pivotal role in regulating food intake and energy homeostasis by acting on the Central Nervous System (CNS) and signaling that adequate energy store are available for the reproduction [53, 54].

Actually, compelling findings have well established that leptin is an extremely versatile hormone which regulates not only body weight and energy homeostasis, but is also involved in immune and neuroendocrine functions and in several other physiological processes such as inflammation, thermogenesis and angiogenesis and reproductive functions [55, 56].

Leptin performs its action by binding to specific leptin receptors (LEPRs), members of the class I cytokine receptor superfamily, with six known isoforms which differ from each other in the size of the intracellular domain and in the tissue expression level [57–59].

LEPR-b, which contains a long intracellular domain, is expressed at high levels in many human brain structures such as hypothalamic nuclei and the cerebellum and is the major isoform responsible for signal transduction of this adipokine [60, 61].

The signaling pathway primarily implicated in leptin signaling is that of JAK/STAT, which involves the activation of Janus kinase 2 (JAK2) and leads to inhibition of the synthesis of many orexigenic neuropeptides, while stimulating the production of some anorexigenic neuropeptides [62]. In detail, this adipokine acts at the level of the arcuate nucleus of the hypothalamus, inhibiting the synthesis of neuropeptide Y (NPY), a neurotransmitter that promotes appetite, and of the oressizing Agouti-related peptide (AgRP); moreover, it also inhibits the production of the hormone concentrating the melanin (MCH) and orexins, powerful appetite promoters [62]. In addition, leptin by binding LEPR-b stimulates neurons to synthesize POMC, the precursor of melanocyte-stimulating hormone-α (α-MSH); the latter is an anorexigenic neuropeptide that decreases food intake. Furthermore, leptin stimulates neurons to synthesize the CART (cocaine-amphetamine-regulated transcript), another molecule capable of inhibiting appetite [62].

In addition to its function in hunger/satiety control, numerous associations have demonstrated the involvement of leptin in reproduction. Specifically, in humans, subjects lacking leptin or functional LEPRs do not reach pubertal maturity and have low serum levels of both FSH and LH [63] indicating the existence of a critical leptin concentration threshold for the onset of the menstrual cycle and the functioning of the reproductive system in women [55, 64].

It also acts as a key regulator and stimulator of the H-P-O axis modulating GnRH secretion in the arcuate hypothalamic neurons in a dose-dependent manner [55]. The regulation of GnRH function in turn regulates gonadotropin secretion and, as GnRH neurons in the preoptic area do not express LEPR-b, GnRH-producing cell bodies are not directly influenced by leptin but by other neuropeptides whose production is influenced by leptin such as NPY, PMOC and kisspeptin, which is an hypothalamic peptide encoded by the KISS1 gene with a key role in regulation of the H-P-0 axis [55, 65]. Specifically, this peptide is widely reported as a key factor in the regulation of LH and FSH secretion and its levels were found to be higher in women with PCOS leading to enhanced H-P-O axis activity, thereby causing irregular menstrual cycles and excessive androgen release in PCOS population [66].

Besides, leptin exerts also effects on the adenohypophysis causing a dose-related increase in LH, FSH and prolactin release through nitric oxide synthase activation [67]. In this regard, several trials have reported that inhibition of LH secretion by dietary restriction was reversed with leptin administration, demonstrating a positive association between LH secretion and leptin [68, 69] and supporting the hypothesis that leptin acts as permissive factor in the development of puberty [70].

LEPRs have been identified in granulosa, theca and interstitial cells of the human ovary. The expression of these receptors in granulosa cells, which synergize with glucocorticoids to promote steroidogenesis, indicates that leptin exerts a direct regulatory action in ovarian folliculogenesis. Furthermore, it has been shown to modulate LH-stimulated estradiol production in the ovary [71].

Circulating concentrations of leptin are generally <10 ng/ml [72] and are influenced by several factors; one of the most important is definitely the amount of adipose tissue. In fact, it has been shown that subjects with obesity have higher leptin levels than subjects of the same age and gender who have a normal weight [73, 74]. Despite an increased level, however, this leptin fails to perform its action adequately; this leads to hypothesize a condition of leptin-resistance, which also involves its receptor. So much so that it has been shown that a chronic increase in leptin levels in the central nervous system leads to a decrease in the expression of LEPRs and in signal transduction [75].

In the pathogenesis of PCOS the role of leptin, and particularly leptin resistance, is not entirely clear but it has been hypothesized that in this disorder the simultaneous presence of insulin resistance and leptin resistance and the interaction between these two hormones is the main one to play an important pathogenetic role.

Confirming this, Hill et al. [19] showed that deletion of leptin and insulin receptors in POMC neurons of female mice resulted in weight gain, insulin resistance, elevated testosterone concentrations, elevated LH concentrations, increased ovarian follicle degeneration, and impaired fertility, all characteristics associated with PCOS.

Furthermore, high leptin concentration in the ovary, is able to suppress estradiol production and interfere with the correct development of dominant follicles and oocyte maturation, predisposing to anovulation. Accordingly, in conditions with excess energy store or metabolic disturbance, as obesity and/or PCOS, leptin has an inhibitory effects on the gonads and induces H-P-O dysfunction [76].

The disturbance of H-P-O axis is suspected to be the main culprit in the development of PCOS and future studies investigating the relationships between leptin and other hormonal and metabolic factors such as LH and IR are needed to debate the role of leptin in the etiopathogenesis of PCOS.

## Laboratory Findings

Taking into consideration the Rotterdam Criteria for the diagnosis of PCOS [9], from a laboratory point of view it is suggested to investigate only biochemical hyperandrogenism. Useful for this purpose is the testosterone assay, the Androstenedione (ANSD) and the Dehydroepiandrosterone Sulfate (DHEA-S) assay but even better the free androgen index (FAI), which is considered more accurate nowadays [77•].

Notwithstanding the above, PCOS being a multifactorial disorder and characterized by different phenotypes [77•], for diagnostic-therapeutic purposes, it would be useful to assay other markers that evaluate not only hormonal but also metabolic and inflammatory alterations, indicative of an impaired crosstalk between adipose tissue and the H-P-O axis.

Dosing of each of the following markers should be considered in light of the clinical picture and the cost:benefit ratio (Table 1).

**Table 1:** Main hormonal, metabolic, and inflammatory laboratory findings useful in the diagnostic-therapeutic pathway of PCOS

MARKER	ROLE	VARIATION	TARGET
Serum Testosterone	Hormonal	↑	Primarily produced by the ovaries. In particular, the free form is elevated in PCOS due to the ↓ in SHBG levels caused by, among the other, obesity and hyperinsulinemia [77•]
Free androgen index (FAI)	Hormonal	↑	Index that more accurately assesses biochemical hyperandrogenism than free testosterone assay, according to the Consensus Conference in Rotterdam [78]
ANSD	Hormonal	↑	Produced by the ovaries and adrenal gland, it undergoes peripheral conversion to estradiol and dihydrotestosterone [77•]
DHEA-S	Hormonal	↑	Produced exclusively by the adrenal gland. Its serum concentration is associated with higher values of testosterone and ANSD and its increase seems to be part of a generalized higher androgen production [79].
LH	Hormonal	↑	Increased pituitary synthesis of LH, as a consequence of persistent GnRH pulsatility, is the stimulus for androgen synthesis by ovarian theca cells and plays an important role in maintaining hyperandrogenism [11]
FSH	Hormonal	=↓	Low but steady FSH levels continuously stimulate the growth of new follicles that fail to reach full maturation, undergoing atresia. These atretic follicles enrich the ovarian stromal portion, which secretes, under the stimulus of LH, a substantial amount of androgens [77•].
LH:FSH ratio	Hormonal	↑	Since FSH secretion would appear to be stimulated by lower GnRH pulsatility, in PCOS the stimulus appears to be in favor of LH resulting in invariance or, more often, reduced FSH synthesis. Thus, higher LH secretion accompanied by equal or lower FSH secretion results in a higher LH:FSH ratio (> 2 in PCOS) [80].
SHBG	Hormonal	↓	Reduced synthesis of SHBG, which in PCOS can also be caused by hyperinsulinemia, leads to a higher concentration of free testosterone and a consequent higher androgenic activity [12]
AMH	Hormonal	↑	Probably due to an intrinsic activation of steroidogenesis and disregulation of granulosa cells function. Excess AMH directly stimulates androgen production and indirectly inhibits the action of FSH on aromatase [77•]. Women with AMH >10 ng/mL had greater prevalence of polycystic ovarian morphology and oligoamenorrhea than women with AMH 5–10 ng/mL [81]
17-OH progesterone	Hormonal	↓	Chronic anovulation, a consequence of failed follicular maturation due to impaired FSH secretion, leads to a reduction in progesterone synthesis [77•]
Estradiol	Hormonal	↑	High estrogen values, which correspond to the physiological early follicular phase, are consistently observed high in PCOS, causing anovulation. They result mainly from peripheral androgen conversion and increased androgen-dependent aromatase activity of ovarian granulosa cells [77•]
Prolactin	Hormonal	=↑	Although there is no precise guidance on the dosing of prolactin levels in women with PCOS, it is well known that estradiol stimulates prolactin secretion. Therefore, hyperprolactinemia could be the result of increased estrogen secretion in patients with PCOS and serve as a reinforcement at diagnosis. An evaluation of prolactin levels in patients with PCOS is recommended, also to identify its causes [82].
Thyroid stimulating hormone, TSH	Hormonal	=↑	In PCOS patients, TSH levels would appear to be higher than in controls. When the TSH level is abnormal, it can adversely affect the hypothalamus and the function of the hypothalamic-pituitary-thyroid axis, and, conversely, the imbalance of the gonadal axis in PCOS patients can affect the pituitary-thyroid axis. Many studies have found that PCOS patients with severe hyperandrogenism and insulin resistance also have abnormal thyroid function [83]
Insulin	Metabolic/Hormonal	↑	It is capable of increasing the frequency and amplitude of secretion of GnRH and LH pulses through upregulation of GnRH gene expression in hypothalamic GnRH neurons. Hyperinsulinemia, which may itself be caused by hyperandrogenism, potentiates the stimulatory effects of LH on androgen production in ovarian theca cells thus contributing to androgen-dependent anovulation, as well as inhibiting the production of SHBG [12, 84]. Insulin is, in addition, a known adipogenic factor.
75 g Oral Glucose Tolerance Test (OGTT)	Metabolic	↑	It is considered the most accurate test for assessing glycemic status in PCOS, regardless of BMI. If OGTT cannot be performed, fasting plasma glucose and/or glycated hemoglobin (HbA1c) should be evaluated, although they are less accurate. An OGTT should be considered in all women with PCOS and without pre-existing diabetes when planning a pregnancy or seeking fertility treatment, given the high risk of hyperglycemia and its associated comorbidities in pregnancy [85].
Homeostatic Model Assessment for Insulin Resistance (HOMA-IR)	Metabolic	=↑	The HOMA-IR is the most widely used index of IR at present and may correlate directly with the severity of PCOS. Useful as part of the overall diagnostic-therapeutic evaluation [86]. In PCOS, insulin resistance can, in addition, induce an inflammatory response that increases NF-κB activation, oxidative stress, and altered TNF-α release from circulating MNCs.
Leptin	Metabolic/Hormonal	↑	It is an adipokine and, in addition to being one of the main regulatory hormones of hunger/satiety control, it also acts as a key regulator and stimulator of the H-P-O axis by modulating GnRH secretion. It can also act at the level of the adenohypophysis by causing increased secretion of LH, FSH and prolactin. Due to the presence of its receptors in granulosa cells and oocytes, it has a regulatory action in ovarian folliculogenesis and can modulate LH-stimulated estradiol production. It also correlates with the amount and distribution of body fat mass and may exert proinflammatory activity [55].
CRP	Inflammatory	↑	Elevated levels of CRP, along with those of other parameters, indicate a state of LGCI in PCOS. In addition, CRP has been shown to be a good marker of inflammation and is one of the most sensitive predictors of cardiovascular morbidity. Importantly, in PCOS, higher CRP levels would appear to be independent of BMI and adiposity [87]
TNF-α	Inflammatory	↑	TNF-α, a pro-inflammatory molecule, appears to be related to hyperandrogenism, increased IR, and excess adiposity, all conditions present in PCOS. In addition, overexpression of TNF-α in muscle and adipose tissues is implicated in the development of IR in humans [88].

## Preventive Strategies and Summary of Current Therapies in PCOS

Due to the complex and multifactorial nature of PCOS, to date, there is no single, definitive therapy, and the preventive and therapeutic strategies currently in use target not only the hallmarks of the disease but also at those hormonal and metabolic alterations that are not diagnostic but typical of PCOS such as IR or excess adiposity.

Preventive strategies are purposed to act on the modifiable causes/effects of PCOS. The Recommendations of the International Evidence-Based Guideline 2023 for the Evaluation and Management of Polycystic Ovary Syndrome [85] suggest recommending that all women with PCOS make lifestyle changes, such as performing regular exercise (physical activity recommendations in women with PCOS do not deviate from exercise guidelines for the general population) and an healthy diet combined with exercise and behavioral strategies, in order to improve body composition, weight management, central adiposity and lipid profile and optimize overall health and quality of life. In this regard, it is widely agreed that there is no specific nutritional strategy (such as the Mediterranean diet, ketogenic diet, or timed-restricted feeding) capable of best and chronically manage all types of PCOS, even if it seems that the Mediterranean diet is the nutritional intervention that has shown the best compliance and yielded the best long-term results in terms of body weight control and metabolism management in PCOS women [77•]. It would be desirable, therefore, for all therapeutic strategies to be agreed with the patient and chosen according to her needs and clinical condition in a tailored setting. For the management of body weight, another therapeutic strategy is represented by bariatric surgery based on the guidelines and recommendations valid for the general population. It promotes significant weight loss, which certainly results in improvement of IR, hyperandrogenism, and ovulatory dysfunction [89]. In some cases it has demonstrated greater efficacy in improving the hormonal and metabolic alterations of PCOS, compared to pharmacological therapy [77•].

In order to curb biochemical and clinical hyperandrogenism, which is one of the most frequent diagnostic criteria and alterations in PCOS, currently the use of Combined Oral Contraceptive Pills (COCPs) is indicated, for at least 6 months, with the aim of suppressing ovulation and stopping cyst formation. Side effects of such drugs, however, include increased risk of thrombotic events and increased production of pro-inflammatory molecules [90].

The use of antiandrogens such as spironolactone and cyproterone acetate, flutamide and finasteride may be considered to treat signs with a high negative psychological impact such as hirsutism and hair loss in women with PCOS, if a non-optimal response occurs following the use of COCPs and/or cosmetic therapy for at least six months [85, 90].

For the treatment of IR, the currently used drug is metformin, which has been shown to improve the metabolic and clinical status of women with PCOS by reducing insulin levels, androgen levels, circulating free Testosterone levels, and increasing SHBG. In addition, metformin acts on adipocytokines such as IL-6, IL-8 and metabolic regulators such as leptin. However, side effects of metformin mainly include gastrointestinal symptoms such as diarrhea, constipation, nausea or other symptoms such as fatigue, dizziness, severe drowsiness, skin and muscle pain, labored breathing, and irregular heartbeat [90].

## Adjuvant Nutraceutical and Pharmacological Treatment

Most of the time, however, current therapeutic strategies are not sufficient to manage the above features, and it is necessary to more precisely treat other impairments resulting from increased adiposity, such as LGCI, hyperandrogenism, IR and leptin resistance, with an adjuvant approach that can be nutraceutical and/or pharmacological (Table 2).

**Table 2:** Main studies on therapeutic strategies for metabolic complications of PCOS

**First Author, Year of publication**	**Study type**	**Therapeutic strategy**	**Results & major findings**
Melo et al., 2022 [91••]	Systematic review	Omega-3	Omega-3 fatty acids supplementation promote beneficial effects in women with PCOS through a significant improvement of the lipid, glycemic and hormonal profile, exerting an anti-androgenic and antioxidant action
Shojaei-Zarghani et al., 2022 [92]	Systematic review	Curcumin	The reviewed animal studies indicated the beneficial effects of curcumin on the management of hormonal and metabolic disturbances in the PCOS condition.
Karimi et al., 2022 [93•]	Systematic review	Resveratrol	Results showed that resveratrol supplementation might be effective in improving PCOS-related symptoms by reducing IR, alleviating dyslipidaemia, improving ovarian morphology and anthropometric indices, regulating the reproductive hormones and reducing inflammation and oxidative stress by affecting biological pathways.
Zhou et al., 2021 [94]	In vivo study	Proanthocyanidins (PCs)	This study demonstrated that PCs have an effect on the regulation of the levels of various hormones and ameliorate ovarian fibrosis in PCOS rats. Moreover, it proposed the involvement of PCs in the inhibition of oxidative stress and the downregulation of the TGF-β1/Smad signaling pathway in PCOS rats.
González et al., 2020 [95]	Cohort study	Salsate	Treatment with salsalate in women with PCOS induced a suppression of lipid- and glucose-stimulated oxidative stress and inflammation manifested by a reduction in ROS generation and a profound decrease in NF-κB activation and TNFα secretion with a improved ovarian hypersecretion of androgens and subsequent induction of ovulation with proper insulin action
Lamos et al., 2017 [96]	Systematic review	GLP-1 RA (Exenatide and liraglutide)	The available studies of GLP-1 RA therapy in the treatment of excess body weight in women with PCOS demonstrate that exenatide and liraglutide are effective in weight reduction either as monotherapy or in combination with metformin. A few studies showed that androgens may be modestly decreased and menstrual frequency may be increased. Eating behavior may be improved with liraglutide therapy. Glucose parameters are generally improved. GLP-1RAs were well-tolerated, with nausea being the most significant adverse side effect. Barriers to utilization may be the short duration studies, lack of familiarity of the medication, the route of administration (injection) and the variable outcomes on ovulation and hyperandrogenism.
Han et al., 2019 [97]	Systematic review and Meta-Analysis	GLP-1 RA	This meta-analysis provides evidence that a GLP-1 receptor agonist is superior to metformin for improving insulin sensitivity. However, no significant difference was shown between the GLP-1 receptor agonist and metformin in terms of improving menstrual frequency and decreasing serum total testosterone and Free Androgen Index. In addition, close attention should be paid to its adverse reactions (e.g. nausea and headache) in clinical applications. Overall, the available evidence is not of high quality, and more multicentre, randomized, controlled studies with a larger sample size and rigorous design are expected in the future to offer more objective clinical data for guiding the clinical treatment of women with PCOS.
Salamun et al., 2018 [98]	Pilot prospective randomized study	GLP-1 RA	Preconception intervention with low-dose liraglutide added to metformin is superior to metformin alone in increasing pregnancy rate in infertile obese women with PCOS, despite comparable weight reduction in both groups. A potential impact of liraglutide on the reproductive system needs further exploration, in particular the GLP-1 impact on endometrial quality and receptivity.
Liu et al., 2017 [99]	Prospective randomized clinical study	Exenatide	In women with PCOS short term treatment with exenatide was linked to significant weight loss and central adiposity reduction, which may further explain the improvements in IR, inflammatory marker and menstrual cycle, which may contribute to increasing pregnancy rates in overweight/obese women
Xu et al., 2017 [100]	Animal study	Empaglifozin (EMPA)	Empagliflozin, a SGLT2 inhibitor, enhanced energy expenditure and attenuated inflammation and IR. Moreover, EMPA suppressed weight gain by enhancing fat utilization and browning and attenuated obesity-induced inflammation and IR by polarizing M2 macrophages in WAT and liver.
Pruett et al., 2021 [101]	Animal study	EMPA	In female Sprague Dawley rats with PCOS, the treatment with EMPA amielorates cardiometabolic abnormalities promoting a beneficial reduction in adiposity, leptin levels and blood pressure.

The following sections will describe the main molecules that could be satisfactory supporting options for the management of metabolic and inflammatory aspects in PCOS, such as polyunsaturated fatty acids (PUFAs) and polyphenols as nutraceuticals and anti-inflammatory molecules, GLP-1 analogs and SGLT2 inhibitors as drugs.

### Nutraceutical Options

#### Omega-3

Polyunsaturated fatty acids (PUFAs) omega(ω)-6 (linoleic acid, LA) and omega (ω)-3 (alpha-linolenic acid, ALA) are essential fatty acids that need to be introduced through the diet. LA is metabolized into arachidonic acid (AA) (20:4ω6) while ALA is metabolized into eicosapentaenoic acid (EPA) (20:5ω3) and docosahexaenoic acid (DHA) (22:6ω3). Due to modern agriculture and modern lifestyle involving more sedentariness and greater consumption of industrial and refined foods, western diets contain excessive levels of omega-6 and very low levels of omega-3, leading to an unhealthy omega-6/omega-3 ratio of around 20:1, instead of 1:1 as was during the evolution of human beings. This causes increased oxidative stress, metabolic alterations, increased body weight, and LGCI, often associated with a higher risk of chronic non-communicable diseases [102]. The Mediterranean diet actually encourages the intake of PUFAs and particularly omega-3 mainly from fish, recommending their consumption at least twice a week [103].

Eicosanoid products derived from omega-6 PUFAs, such as prostaglandin (PG) E2 and leukotriene (LT) B4, synthesized from AA, are very potent mediators of thrombosis and inflammation. AA leads to insulin resistance, central leptin resistance, and hepatic steatosis. It also interferes in the proper functioning of the PI3-Akt pathway, involved in both glucose and lipid metabolism, as demonstrated by Cheng et al. [104]. In the hypothalamus, the induction of the PI3K pathway can be regulated by leptin. In fact, leptin induces PI3K through the phosphorylation of Akt and FOXO1 [104] in POMC neurons while leptin withdrawal activates PI3K in AgRP neurons [105]. A disruption of the PI3K pathway can lead to leptin resistance in the CNS and dysregulation of food intake. It has been hypothesized that AA affects this pathway through a combined effect on both leptin and insulin, since both insulin and leptin regulate energy homeostasis through the PI3K-Akt signaling pathway. However, further studies are needed to confirm this hypothesis [104]. Thus, an omega-6/omega-3 ratio shifted toward omega-6 is highly prothrombotic and proinflammatory and contributes to the prevalence of atherosclerosis, obesity, and TD2 [106]. In fact, regular consumption of omega-3-rich diets has been associated with a low incidence of these diseases [107–109].

Kabir et al. [110], instead, assayed the effects of omega-3 supplementation, for 2 months, in a population of women with TD2 and found a significant reduction in total body fat mass and adipocyte diameter in the subcutaneous tissue, that was associated with a reduction of the expression of a whole range of inflammation-related genes.

In PCOS, omega-3 fatty acids consumption may be a promising nutritional strategy for adjuvant treatment of the disease due to its antiatherogenic and anti-inflammatory effects. Also, a very recent review [91••] reported that supplementation with omega-3 fatty acids of animal or vegetal origin could have indirect beneficial effects on PCOS through significant improvement in lipid and glycemic profiles, as well as some effects on hormonal profile, with probable anti-androgenic effect, and on antioxidant and inflammatory markers.

This relationship was well demonstrated in a study that observed the omega-6/omega-3 ratio in the blood plasma of women with PCOS and concluded that the higher the omega-6/omega-3 ratio in the plasma, the higher levels of testosterone and DHEA-S [111]. The omega-3 supplementation of 1.9 g (EPA + DHA) for 6 weeks can reduce free testosterone levels without interfering with the total testosterone, DHEA-S, androstenedione, SHBG, estrogen, LH or FSH. Similarly, the omega-3 supplementation of 800 mg for 12 weeks or 1500 mg for a period of more than 6 months significantly reduced several steroidogenic parameters, such as serum levels of total testosterone, free testosterone, LH and hirsutism score. Lower doses of EPA + DHA (600 mg/day) did not reduce the hirsutism index, but the menstrual cycle of women with PCOS became more regular [91••].

The anti-inflammatory effect of PUFAs is also expressed by Specialized Pro-Resolving Lipid Mediators (SPMs), which are proper terminally active metabolites derived from PUFAs. In animal and in vitro models of adiposity, SPMs have been shown to be able to reduce the levels of pro-inflammatory cytokines such as IL-6, TNF-alpha, and IFN-gamma and increase those of IL-10, which has anti-inflammatory activity [112]. In addition, SPMs would appear to be able to counteract adipokine secretion and monocyte accumulation, promote M2 polarization of macrophages, and improve insulin sensitivity [113].

In addition, several in vivo studies in rats and mice reported that prolonged intake of high omega-3 diets was accompanied by significant decreases in plasma leptin levels, probably secondary to observed decreases in white adipose tissue mass [114, 115]. In rats and mice, n-3 PUFAs supplementation through a high-fat diet reduced plasma leptin concentration, counteracting hyperleptinemia, reduced leptin mRNA expression in adipose tissue, and altered gene expression of brain satiety regulators. In fact, in the mice study, the addition of omega-3 to the diet counteracted both increased expression of leptin, LEPR, and POMC and decreased expression of NPY, whose mRNA levels were assessed by RNA extracted from fat and mouse brain [116].

#### Polyphenols

Several nutraceutical substances have demonstrated effective properties in ameliorating a number of metabolic-related PCOS alterations, such as LGCI, IR, hyperandrogenism, or leptin resistance [117••].

Among these nutrients it is worth mentioning curcumin, which has known anti-inflammatory properties and has been shown to lower leptin levels in both overweight and obese humans [118]. Many studies have assayed the effects of curcumin on the management of hormonal and metabolic disturbances and complications in animal models of PCOS, providing non-unique data due to the different formulations, doses and bioavailability of the molecule used in the different protocols [92].

Improvement in hormonal disturbances, glycemic control, lipid profile, anthropometric characteristics, oxidative stress and inflammation in animals with PCOS, treated with curcumin, was reported in some randomized-controlled trials RCTs considered in a recent review [92].

Another recent report [119•] has already surveyed many of the possible nutrients and bioactive foods that have demonstrated hypoleptinizing activity.

The main ones include polyphenols, found mainly in fruits and vegetables, and, in particular, resveratrol present in grapes, which seems capable of lowering plasma leptin levels by inducing a reduction in p-STAT3 in the hypothalamus [120, 121] being able to cross the blood-brain barrier [122].

In a recent systematic review [93•], which considered both in vitro studies and animal or human models of PCOS, it has been highlighted that resveratrol supplementation could be effective in reducing IR, improving dyslipidemia, ovarian morphology as well as anthropometric indices, regulating reproductive hormones and reducing inflammation and oxidative stress, influencing biological pathways.

Similarly to resveratrol, also proanthocyanidins (PCs), oligomeric repeats of flavonoids obtained from pine bark and grape seeds, have also shown an effect in reducing food ingestion by increasing POMC mRNA levels and improving both central and peripheral leptin sensitivity and LGCI through normalization of the levels of STAT3 [120]. Furthermore, Zhou Y. et al. [94] wanted to assay the effect of PCs in a rat model of PCOS, observing that PC administration significantly reduced body weight (BW) and relative ovarian weight, serum Testosterone level, LH and LH/FSH ratio in the PCOS group. In addition, their results also revealed that PC treatment significantly increased major antioxidant enzymes (Cat, Sod2, Gpx3, Mgst1, Gsta4, Sod1 and Prdx3) in PCOS rats.

Another phenolic compound, oleuropein found in olives and virgin olive oils, supplemented in obese mice, showed regulatory effects on leptin mRNA levels in epididymal adipose tissue and reduced serum leptin levels as compared to lean mice [123]**.** In addition to demonstrating efficacy in reducing oxidative stress and hyperglycemia in diabetic rabbits [124].

There are no known studies on this substance in models of PCOS but knowing its effects on features also present in this disease, its use within a combined therapeutic strategy is auspicable [124].

### Pharmacological Options

#### Anti-Inflammatory Molecules

Treatment options for LGCI include many possible molecules with an anti-inflammatory activity that could be enumerated in both the pharmacological and nutraceutical fields. Since we are referring to a milieu of molecules and pathways that are numerous and complex, as many are possible therapeutic targets.

As mentioned before, it is well known that molecules involved in inflammation play a substantial role in the regulation of ovarian function and any alterations in their levels can lead to ovarian disruption, suggesting their active role in the pathogenesis of PCOS [39].

Knowing in detail what these molecules are and how they act could chart a further course for the treatment of PCOS.

Actually, for the treatment of LGCI in patients with PCOS, common anti-inflammatory drugs such as Non-Steroidal Anti-Inflammatory Drugs (NSAIDs) do not seem to be the first-line therapy. In fact, their prolonged use is known to worsen wound healing, increase the risk of osteoporosis, and may cause gastrointestinal bleeding, as well as increase cardiovascular risk [125].

A clinical trial designed to evaluate the effect of salsalate administration on ovarian androgen-secreting capacity and insulin sensitivity in 90 women with PCOS is currently ongoing; forty-five subjects with PCOS (15 lean without IR), 15 lean with IR, and 15 obese) receiving salsalate, a nonacetylated salicylate, at an oral dose of 3–4 g per day for 12 weeks will be compared with 45 control women with PCOS matched for age and body composition receiving placebo [95]. The rationale of the study is based on the fact that non-acetylated salicylates suppress the activation of NFĸB involved in inflammation and that, in PCOS, suppression of oxidative stress and inflammation induced by salsalate would appear to be associated with improved ovarian hypersecretion of androgens and subsequent induction of ovulation with proper insulin action [95].

Furthermore, the use of therapies targeting TNF signaling has also been hypothesized, but these lead to an increased risk of infections, lymphomas, and non-melanoma skin cancers [112].

For these reasons, in recent years, the field of research has focused on the study of nutraceutical substances, in addition to the aforementioned omega-3 s.

### GLP-1 Analogs

Other molecules that are being studied and are providing good results for the treatment of PCOS affected patients with metabolic disturbance are Glucagon-like Peptide-1 Receptor (GLP-1R) analogs. GLP-1 Rs are a class of drugs primarily used as oral hypoglycemic agents in individuals with diabetes, given their incretin-mimetic activity. Incretins (such as Gastric Inhibitory Peptide GIP and GLP-1) are intestinal hormones secreted by enteroendocrine cells that promote insulin secretion by the pancreas in response to ingested food [126].

Currently used in the treatment of T2D they improve glycemic control and insulin resistance, promote weight loss and have neuroprotective and anti-inflammatory effects [127].

To date, between 2014 and 2015, both the Food and Drug Administration (FDA) and the European Medicines Agency (EMA) have approved liraglutide 3.0 mg/day subcutaneously for the treatment of obesity with BMI ≥ 30 kg/m2 or overweight individuals with BMI ≥ 27 kg/m2 with other risk factors or other obesity-related conditions. Semaglutide 2.4 mg/week was approved, by FDA in June 2021 and by EMA in November 2021, with the same indications, yielding exciting results.

Furthermore, human studies have demonstrated the efficacy of GLP-1 analogs in reducing BMI and waist circumference and improving glycemic profile and IR in women with PCOS compared with metformin alone; even more promising data were obtained when combination therapy with GLP1 analogs plus metformin was tested, as compared with metformin alone [128••]. The mechanisms by which these agents contribute to increased insulin sensitivity and improved glucose homeostasis are complex and still under investigation. It has been suggested that GLP-1 agonists may act both on pancreatic insulin secretion and through a series of extrapancreatic actions. GLP-1 R analogs therapy increases plasma GLP-1 and increases glucose-dependent insulin secretion, reduces glucagon secretion, slows gastric emptying, and stimulates satiety [96]. Other studies in animal and human models of PCOS, which assayed the effects of GLP-1 analogs both alone and compared with metformin, also provided pleasing results in reducing androgen levels [99]. The mechanisms underlying this activity are still not entirely clear and the results of the studies are not unanimous so further investigation is needed in this regard.

In addition, they may modulate molecular pathways involved in inflammation, oxidative stress, lipid metabolism, β-cell function, and insulin activity [129].

The effects of GLP-1 analogs on fertility have been most often investigated in animal models of PCOS and insufficiently in humans, yielding satisfactory results.

However, Salumun et al. [98] and Liu et al. [99] evaluated the effects of treatment with two types of GLP-1 analogs. Specifically, they administered liraglutide (1.2 mg daily) plus metformin and exenatide (10 μg twice daily), compared with metformin alone, respectively, on spontaneous pregnancy rates, observing in both cases significantly better results in the treated group [128••].

In conclusion, given the importance of these effects and the actions of GLP-1 analogs with positive results also in improving hyperandrogenism and oligo/amenorrhea, these pharmaceutical agents could be crucial in the treatment of PCOS [96].

### SGLT2 Inhibitors

Among the SGLT2 inhibitors, canaglifozin was the first one approved in 2013 by the FDA for the treatment of T2D. They act by inhibiting glucose reabsorption in the proximal portion of the renal tubule, resulting in glycosuria. As a result, they lower blood glucose levels through an insulin-independent mechanism, as well as reduce hyperinsulinism. [130].

The possible use of SGLT2 inhibitors in PCOS is justified by the fact that in addition to acting on glycemic control, they have shown efficacy in improving other typical aspects of this pathology such as excessive body fat mass, LGCI and the dysregulated production of the main adipokines such as leptin, in addition to reducing oxidative stress, blood pressure and so protecting the cardiovascular system [101].

Studies in animal models have mainly investigated the effect of empaglifozin (EMPA) on LGCI and other PCOS-related dysmetabolisms. Xu and colleagues [100] used obese mice induced by high-fat diets as a study model, showing that empagliflozin was able to increase energy expenditure and attenuate inflammation and insulin resistance. The first result was achieved by increasing the expression of uncoupling protein 1 in brown fat and white adipose tissue (WAT) inguinal and epididymal, resulting in reduced body weight of treated mice. For the second point, it would appear that empagliflozin succeeds in reducing the accumulation of M1-polarized macrophages while inducing the M2 anti-inflammatory phenotype of macrophages in WAT and liver, lowering plasma TNFα levels and attenuating obesity-related chronic inflammation.

The latter finding was also confirmed in the study by Pruett and colleagues [101], in which it was also shown that the reductions in adipose tissue given by EMPA treatment are associated with a decrease in plasma leptin. Leptin is capable of stimulating the sympathetic nervous system, among other actions, and, as we discussed earlier, its increase is associated with PCOS. Therefore, the decrease in leptin may have led to less activation of the sympathetic nervous system, which may partially explain another important finding highlighted in this study, namely the decrease in blood pressure in EMPA-treated PCOS rats.

In women with PCOS, comparative trials have been conducted mainly between metformin (or placebo) and an SGLT2 inhibitor showing results supporting the latter on both hormonal, metabolic and anthropometric parameters [84].

## Conclusion

Polycystic ovary syndrome (PCOS), which is the major endocrine-metabolic disorder of women’s reproductive age, can be considered the prototypical disease in which the effects of the relationship between adiposity and hypothalamic-pituitary-ovarian (H-P-O) axis are made explicit.

Already in pre-pubertal age, but even more so in adulthood, the association between visceral obesity and ovulatory disorders secondary to neuroendocrine alterations of the H-P-O axis has been demonstrated. Indeed, as in a self-feeding cycle, elevated androgen levels in PCOS lead to visceral fat deposition, resulting in hyperinsulinemia, IR, hyperleptinemia, and increased LGCI, further stimulating ovarian and adrenal androgen production, which, produced in excess, are aromatized into estrogen at high rates in the periphery, causing negative feedback on the H-P-O axis and affecting gonadotropin production. In particular, the persistently rapid pulsatility of GnRH, which promotes increased pituitary synthesis of LH acts as a stimulus for androgen synthesis by the theca cells. All this results in menstrual abnormalities and ovulatory dysfunction.

These alterations can both directly or undirectly adversely affect the physiological function of the H-P-O axis.

Nowadays, the world of medicine and therapy is moving more and more toward precision medicine and personalized treatments, so our intent to explore aspects beyond the canonical hormone therapies and good health practices for PCOS treatment meets the advancement in the field. This certainly includes exercise and healthy nutritional treatment with the Mediterranean diet, which seems likely to be the most successful long-term nutritional treatment with regard to compliance and management of metabolic disorders and treatment with Combined Oral Contraceptive Pills (COCPs), antiandrogens, and/or metformin, as appropriate. The latter can be assisted by either nutraceutical or pharmacological adjuvant therapy in order to also better manage other hormonal and metabolic features of PCOS.

In fact, it has been shown in both animal and human studies that supplementation with omega-3 PUFAs has beneficial effects on both hyperandrogenism and insulin resistance, hyperleptinemia, and especially LGCI for their known anti-inflammatory properties. Polyphenols such as resveratrol, proanthocyanidins, or oleuropein may also can contribute to the improvement of metabolic changes typical of PCOS.

GLP-1 analogs, which originated for the treatment of diabetes, are proving to be extremely effective for the treatment of obesity and for regulation of molecular pathways involved in inflammation, oxidative stress, lipid metabolism, insulin activity, as well as androgen synthesis, gaining promising results in animal studies testing their efficacy on spontaneous pregnancy rates.

Last but not least, PCOS, in addition to being one of the most frequent reproductive disorders, can also be considered a cardiometabolic disorder with cardiovascular risk, which is reportedly increased in women with PCOS and therefore should not be neglected. Hence, the possible use of SGLT2 inhibitors in PCOS which, originated as molecules for the treatment of Diabetes Mellitus type 2,in addition to acting on glycemic control, have shown efficacy in treating excess body fat mass, LGCI and the consequent dysregulation of the production of major adipokines such as leptin, but especially in reducing oxidative stress, blood pressure and to have, therefore, a protective effect on the cardiovascular system.

In the light of the above, especially in multifactorial diseases such as PCOS, it is desirable to choose a therapeutic strategy that is as tailored as possible, based on the clinical setting and the needs of the specific patient, going to detect and, therefore, also treat the side alterations of the disease, as an adjuvant treatment to the canonical therapy, in order to maximize therapeutic intervention and take advantage of any synergistic effects among the various approaches.

## Abbreviations

AA: Arachidonic Acid
AgRP: Agouti-Related Peptide
AKT: Protein Kinase B
ALA: Alpha-Linolenic Acid
BMI: Body Mass Index
CNS: Central Nervous System
COCPs: Combined Oral Contraceptive Pills
CRP: C-reactive protein
DHA: Docosahexaenoic Acid
DHEA-S: Dehydroepiandrosterone Sulfate
EPA: Eicosapentaenoic Acid
FOXO1: Forkhead Box O 1
FSH: Follicle-Stimulating Hormone
GLP-1R: Glucagon-like Peptide-1
GLUT-4: Glucose Transporter Type 4
GnRH: Gonadotropin-Releasing Hormone
HOMA-IR: Homeostatic Model Assessment for Insulin Resistance
H-P-O: Hypothalamic-Pituitary-Ovarian
IFN-gamma: Interferon-gamma
IGF-1: Insulin-like Growth Factor-1
IL-10: Interleukin-10
IL-17: Interleukin-17
IL-23: Interleukin-23
IL-6: Interleukin-6
IR: Insulin Resistance
IRS: Insulin Receptor Substrate
JAK: Janus Kinase
LA: linoleic acid,
LEPRs: Leptin Receptors
LH: Luteinizing Hormone
LT: Leukotriene
NF-κB: Nuclear Factor-κB
NPY: Neuropeptide Y
NSAIDs: Non-Steroidal Anti-Inflammatory Drugs
OGTT: Oral Glucose Tolerance Test
PCOS: Polycystic Ovary Syndrome
PCs: Proanthocyanidins
PG: Prostaglandin
PI3K: Phosphatidylinositol 3-Kinase
POMC: Proopiomelanocortin
PUFAs: Polyunsaturated Fatty Acids
RCTs: Randomized-Controlled Trials
SGLT2: Sodium-Glucose Transport Protein 2
SHBG: Sex Hormone Binding Globulin
SPMs: Specialized Pro-Resolving Lipid Mediators
STAT: Signal Transducer and Activator of Transcription protein
T2D: Type 2 Diabetes
TGF-β: Transforming Growth Factor-β
Th17: T-helper 17
TNF-α: Tumor Necrosis Factor-α
α-MSH: Melanocyte-Stimulating Hormone-α
